# Rapidly dissolving microneedle patch of amphotericin B for intracorneal fungal infections

**DOI:** 10.1007/s13346-021-01032-2

**Published:** 2021-07-23

**Authors:** Alyaa A. Albadr, Ismaiel A. Tekko, Lalitkumar K. Vora, Ahlam A. Ali, Garry Laverty, Ryan F. Donnelly, Raghu Raj Singh Thakur

**Affiliations:** 1 School of Pharmacy, Medical Biology Centre, Queen’s University Belfast, 97 Lisburn Road, Belfast, BT9 7BL Northern Ireland UK; 2 Biology Department, Science College, Basra University, Basra, Iraq; 3 Faculty of Pharmacy, Aleppo University, Aleppo, Syria

**Keywords:** Amphotericin B, Intracorneal, Microneedles, Biocompatibility, Hyaluronic acid, Poly (vinylpyrrolidone)

## Abstract

Chronic fungal infection of the cornea could lead to blindness if not treated properly. Topical amphotericin B (AMP-B) is considered the first treatment of choice for ocular fungal infection. However, factors related to its poor solubility and penetration through intact cornea lead to poor bioavailability. Microneedles (MNs) are emerging as a minimally invasive method to enhance ocular drug delivery. This study aims to investigate the potential use of biodegradable poly(vinylpyrrolidone) (PVP) and hyaluronic acid (HA)–based rapidly dissolving MNs for delivery of AMP-B to treat fungal infection. The data obtained illustrates PVP/HA MN arrays’ reproducibility, good mechanical strength, and faster dissolution with 100% drug recovery. Multiphoton microscopic results revealed that MNs successfully penetrate the corneal tissue and enhance AMP-B permeation through corneal layers. Furthermore, PVP/HA MN arrays showed high solubility. Both PVP and HA successfully decreased AMP-B cytotoxicity when compared to free drug. More interestingly, the biocompatible MN formulations preserved the antifungal activity of AMP-B, as demonstrated by significant inhibition of fungal growth. Therefore, this study shows the feasibility of ocular delivery of the poorly soluble AMP-B using a fast-dissolving MN patch.

**Graphical abstract:**

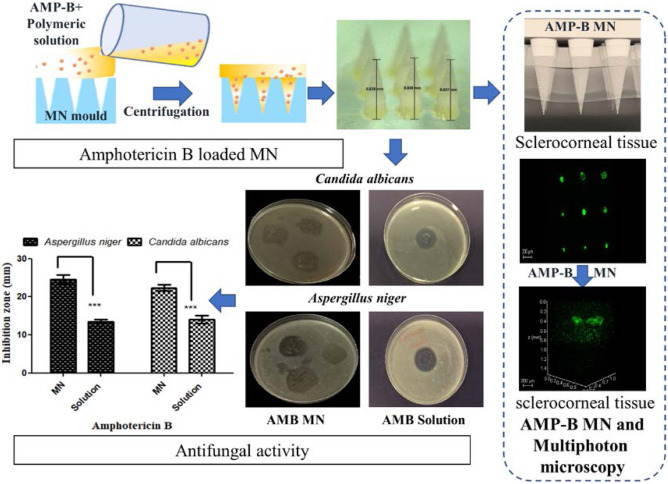

## Introduction

The cornea is a transparent layer covering the front of the eye and plays a vital role in protection against several abiotic factors like fine hairs and sand, and biotic factors such as bacteria, fungi and viruses [1]. Ocular fungal infection is a microbial disease caused by various fungi such as *Candida*, *Aspergillus* and *Fusarium* species [2]. According to a recent publication, around 1 million fungal keratitis cases have been estimated yearly [3]. Treatment of fungal infection is challenging because of anterior ocular barriers like corneal epithelium and tear fluid, limiting drug availability and the lack of efficient drug delivery systems [4]. A limited number of antifungal drugs (azoles, polyenes and echinocandins) are currently used to treat fungal infections [5]. However, both azoles and echinocandins groups develop drug resistance, but to a lower extent than polyene’s antifungal drugs such as amphotericin B (AMP-B) [6].

AMP-B was discovered seven decades ago as a secondary metabolite from soil species of actinomycetes called *Streptomyces nodosus* and widely used to treat fungal infections either intravenously or topically [7–9]. The antifungal activity of AMP-B is attributed to its ability to obstruct the synthesis of the fungal cell wall by binding with sterol molecules forming an ergosterol-AMP-B complex that leads to pore formation and leaking of intracellular ions, which eventually causes fungal cell destruction and death [7].

The conventional formulation of AMP-B is AMP-B deoxycholate (Fungizone®), commonly administered as an injection and suffers from limited dose toxicity such as infusion-related reaction and nephrotoxicity, and this often prevents further administration leading to incomplete treatment course [8]. Typical formulations such as eye drops of AMP-B provide inadequate drug absorption and poor corneal bioavailability [9]. Ocular administration (subconjunctival injection) also showed some toxicity which was attributed to the presence of deoxycholate [10].

In an attempt to improve the therapeutic index of AMP-B, different lipid formulations were developed, such as AMP-B lipid complex (ABLC), liposomal AMP-B (L-AmB), and AMP-B colloidal dispersion (ABCD) [11]. Although lipid formulations were shown as safer alternatives to conventional aqueous AMP-B formulations, localised administration of AMP-B lipid formulations was shown to induce toxicity due to the presence of phospholipids that cause pseudo-hyperphosphatemia [12].

Drug delivery systems using biodegradable polymers were emerged to increase solubility and reduce drug toxicity. Biodegradable polymers could potentially improve local residence time, prolong drug action and improve bioavailability [13–15]. Incorporation of biodegradable, biocompatible polymers including oxidized arabinogalactan, polyvinylpyrrolidone (PVP), polyvinyl alcohol (PVA), copolymer polylactic acid and polyglycolic acid (PLGA)-TPGS (tocopheryl polyethylene glycol succinate) shown in vitro and in vivo decrease in toxicity of AMP-B [16, 17]. However, it was noticed that all previous studies were devoted to developing AMP-B formulations for systemic administration, orally [18, 19] or intravenously [20, 21]. Limited studies demonstrated the use of topical AMP-B formulations with biodegradable polymers for ocular delivery. For instance, collagen was used as a shield of contact lenses for releasing AMP-B on the cornea’s surface [22]. Furthermore, chitosan was incorporated in poly (lactic acid) to produce self-assembled nanoparticles for ophthalmic delivery of AMP-B [23].

Microneedles (MNs) have emerged as a practical approach for controlled drug release locally [24]. This promising technique was successfully employed for improving drug delivery through the skin [25–28] and further developed as an efficient approach for ocular delivery by our group [29, 30]. MNs are minimally invasive micron-sized needles ranging from 60 to 2000 µm in height and varying arrays [31, 32], and economically cheaper than other delivery strategies. For example, designing and producing a single biodegradable MN for medical use with AMP-B could be more affordable, whereas advanced liposomal formulations for AMP-B cost around $85 per 50 mg/vial [33].

We have previously demonstrated the use of dissolve PVP MNs to improve ocular drug delivery of large model molecules (fluorescein isothiocyanate-dextran) by disturbing the cornea’s barrier function sclera [29, 30]. Similarly, recent studies by Bhatnagar et al. [35] showed that delivery of besifloxacin loaded in PVP/PVA MNs successfully treated bacterial keratitis. Also, Sachan et al. [34] demonstrated polyglycolic acid MNs for transdermal delivery of AMP-B. Roy et al. [35] fabricated liposomal AMP-B to load into the polymeric MNs. However, liposomal formulation reduced the loading capacity in micron-sized MN tips, and high loading of lipidic liposomal formulation could affect the mechanical strength of MN adversely. Additionally, increasing MN arrays or patch size for the corneal application can cause corneal scarring. Therefore, this study aims to investigate to directly load AMP-B in biodegradable PVP/PVA and hyaluronic acid (HA)–based rapidly dissolved polymeric MNs to treat ocular fungal infection via intracorneal route. The MNs were thoroughly characterized for their mechanical strength, dissolution characteristics, insertion depths in the artificial membrane and corneal tissues. Multiphoton microscopic studies were conducted to investigate the depth of AMP-B penetration and distribution within the corneal tissues. In vitro antifungal activity of AMP-B encapsulated MNs and biocompatibility of the polymers was also investigated.

## Materials and methods

### Materials

AMP-B was purchased from Cayman Chemical (Michigan, USA). Polyvinyl pyrrolidone (PVP) K29–32, MW 58,000 Da, was gifted from Ashland Inc. (Surrey, UK). Hyaluronic acid (sodium hyaluronate 200-400 K) (HA) was purchased from Kewpie Corporation (Tokyo, Japan). Polyvinyl alcohol (PVA), 85,000–124,000 Da, was purchased from Sigma–Aldrich (Dorset, UK). ARPE-19 cells were purchased from the American Type Culture Collection (ATCC). Dulbecco’s modified Eagle’s medium/F-12 human amniotic membrane nutrient mixture (DMEM) was purchased from Life Technologies (UK). Fetal bovine serum (FBS) and trypsin were purchased from Life Technologies (UK). 3-(4,5-dimethylthiazol-2-yl)-5-(3-carboxymethoxy-phenyl)-2-(4-sulfophenyl)-2H-tetrazolium, inner salt (MTS) agent was purchased from Promega (UK). Fungal isolates including *Candida albicans* (NCTC 3179) and *Aspergillus niger* (CABI 017,454) were kindly donated by Dr Fiornuala Lundy, Queen’s University, Belfast, School of Medicine, Dentistry and Biomedical Sciences, Centre for Infection and immunity.

### Methods

#### Preparation of hydrogels for MN fabrication

A range of polymers was used to fabricate dissolving MNs, as summarised in Table 1. The hydration process of these polymers was performed, according to Thakur et al. [29]. Briefly, polymer powder was mixed with deionised water and vortexed for 1 min. Then, the polymer solution for PVP and HA was sonicated for 1–2 h at 37 °C. PVA solution was heated to 90 °C and mixed on a magnetic stirrer for 2 h. Polymer stock solution was stored at ambient temperature overnight for complete hydration. The final concentration of each polymer was adjusted by adding the rest of the deionized water and mixed by vortex for homogenising. The homogenised gels were used for the fabrication of MNs.

**Table 1:** Composition of different hydrogel formulations used in MN fabrication

Formulation code	Polymer (w/w)
F1	20%PVP
F2	30%PVP
F3	5% HA
F4	30% PVP + 7.5%PVA
F5	30%PVP + 10%PVA
F6	30%PVP + 15%PVA
F7	15% PVP + 15% PVA
F8	20% PVP + 5% HA
F9	30%PVP + 5% HA

#### Polymeric MN fabrication

Silicone MN moulds were used for creating MN arrays of 3 × 3 polymeric microneedles which have a conical shaped with dimensions H: 750 µm × W: 300 µm, and an interspacing among needles equals 300 µm. Around 50 mg of hydrogel formulation with or without AMP-B was placed into silicone moulds and centrifuged for 15 min at 5000 rpm to ensure complete filling of the MN cavities. MNs were dried at room temperature for 48 h. Then, MN arrays were carefully removed from the silicone moulds. Dried MN was kept in a closed container with aluminium foil covering in the refrigerator until further use for characterisation.

#### Microneedle characterisation

##### Drug content determination

MN arrays were delicately removed from the silicone moulds and put in a 20 mL vial to dissolved in 10 mL PBS by a magnetic stirring at 400 rpm for 30 min. Centrifugation was performed at 5000 rpm for 10 min to remove any aggregated particles. A 100 µL of supernatant was serially diluted to be its absorbance value readable via UV–visible spectroscopy and defined by calibration curve range. Data were described as drug weight in µg per 3 arrays (mean ± SD, *n* = 3).

##### Determination of mechanical properties of MNs

Mechanical strength of polymeric shafts of MN arrays was determined according to Thakur et al. [29] by applying a compression test that includes using different predetermined forces generated by texture analyser (Stable Microsystems, Haselmere, UK). Briefly, before testing, the MN arrays were visualised by a Leica MZ6 dissection microscope (Leica Microsystems UK Ltd., Milton Keynes, UK). The heights of MN shafts were recorded for comparison with the heights of the shafts after the compression test. The MN array, in a downward direction, was carefully put on a plane stainless steel platform of the probe of the texture analyser. The probe was moved down at a speed of 0.1 mm/s for making MNs touched a flat stainless steel solid bulk, and its predefined forces were used. The probe stayed at the predetermined force for 30 s. After that, the probe was moved upward at a speed of 1 mm/s. The MN were examined visually by the light microscope to determine the heights of arrays after compressing test. The rate of changes of the MN height (µm) was calculated and plotted as the reduction percentage in MN height against applied forces. The experiment was performed in triplicates. 

##### MN dissolution test

The time needed for the dissolving of the arrays of polymeric MNs was determined using the porcine corneal tissue as dissolution medium. The circular pieces of corneal tissues were prepared by using a sharp disposable scalpel; then, these tissues were hydrated by placing them in PBS at ambient temperature 23 ± 2 °C for 2 h. The corneal tissues were delicately dried using clean tissues and fixed to a customised circular sheet made from a weighing boat utilising cyanoacrylate glue (Loctite Ltd., Dublin, Ireland). Then, the fixed sclerocorneal tissues were put on board of dental wax for supporting the tissues. The MN arrays were then applied with gentle pressure to insert into the middle of the corneal tissue by customised handle tool and grasped there for predetermined time periods (0, 30, 60, 90, 120, 150, 180 s). After that, the arrays of MN were envisioned using a Leica MZ6 dissection microscope fitted with a Nikon Coolpix 950 digital camera (Nikon UK Ltd., Surrey, UK). Before and after insertion, the heights of MNs were measured, and the reduction of MN height percentage was calculated and recorded as remaining MN height (µm) versus time (s). Each experiment was performed in three replicates. 

##### Insertion test of MN arrays into parafilm M® as a corneal simulant

According to Larrañeta et al., [36] this test was performed using a sheet of polymeric film (Parafilm M®) (Bemis NA, USA). Polymeric MN arrays were placed on the Parafilm M® layers and fixed carefully on the plane surface. The aluminium probe of the texture analyser was lowered toward the Parafilm M® at a speed of 0.5 mm/s till the specified force was applied for 30 s. Several forces were held from 0.2 to 5 N per array. After reaching the target force, the probe was changed its position upwards using the same previous speed of 0.5 mm/s. The number of visible holes produced by the arrays of MNs was counted, and the following equation was applied to present the relationship between the tested force and parafilm M® penetration [37].

$$\begin{aligned}Percentage \;of\; &MN\; arrays\; penetration\;\\&\qquad=\frac{ number \;of\; holes\; observed }{number \;of \;holes\; expected }\times 100\end{aligned}$$

##### The Parafilm M® insertion depth measurement using OCT

According to the rapid dissolvable nature of MN materials, it is difficult to check the insertion depth measurement in cornea tissue. Therefore, the determination of MN depth penetration was carried out on the layers of artificial membrane (Parafilm M®) with the same thickness as cornea tissue. To facilitate visualisation and measurement of the depth of the microscopic needles into the layers of the artificial membrane, optical coherence tomography (OCT) (EX1301 OCT microscope, Michelson Diagnostics, Kent, UK) was employed. AMP-B-loaded MNs were inserted into the folded Parafilm M® layers using the same range of forces (0.2 to 5 N) that were applied by texture analyser. After the penetration process of MN arrays into Parafilm M®layers, the swept source/Fourier domain OCT at 1305 ± 15 nm was employed to aid real-time high-resolution imaging of the surface of the artificial membrane layers with 7.5 μm lateral resolution and 10.0 μm for vertical. The MN inserted layers of Parafilm M® were scanned at a frame rate of up to 15 B-scans (two-dimensional cross-sectional scans) per second. The scan width was 2 mm. The analysing of the 2D images was performed by the imaging software ImageJ® (National Institutes of Health, Bethesda, USA). The percentage of insertion was calculated depending on the difference between the initial length of the MN shafts before inserting and the length of the shafts inside the artificial folded membrane layers after exposure to specified forces previously.

$$\begin{aligned}&Percentage \;of \;MN \;arrays \;insertion\;\\&\;\;\;\;=\;\frac{length \;of \;the\; microneedles \;after \;insertion }{length \;of\; the \;microneedles\; before\; insertion }\times 100\end{aligned}$$

#### Preparation of ocular tissues

Porcine eyeballs were used to obtain ocular tissues because of histological similarity between the human eye and the pig eye [38]. The porcine eyeballs were collected from a local slaughterhouse and stored in a freezer at − 80 °C until use. The sclerocorneal tissue was isolated from other eyeball parts. It was washed many times by sterile phosphate buffer solution (PBS pH 7.4), and then dried using fine tissue, and stored in a sealed container at − 80 °C until further use.

#### Fungal inoculum suspension

The inoculum size was adjusted for all fungal isolates according to the McFarland scale. Turbidity was adjusted to a 0.5 McFarland turbidity using a spectrophotometer at 530 nm (CO7500 Colorimeter). Densities were 1.5–5 × 10^6^ CFU/mL for *Candida albicans*, and 2–3 × 10^4^ CFU/mL for *Aspergillus niger*. Those concentrations were used as standard inoculum in all experiments.

#### In vitro studies

##### Intraocular AMP-B delivery and distribution studies using multiphoton microscopy

The corneal tissue with thin rims of sclera tissue was used to study AMP-B distribution by multiphoton microscopy. Three groups were used to perform this study: (1) treated sclerocorneal tissue with a topical application of 200 µL of AMP-B suspension (1000 µg/mL), (2) sclerocorneal tissue treated with MN-loaded AMP-B, and (3) sclerocorneal tissue free from AMP-B administration. Sclerocorneal tissue of each group was prepared previously, and each one was placed on the microscope glass slide on the Leica upright microscope stand DM6000 (Leica Microsystem, Milton Keynes, UK). The images were taken for each treatment, including control, using Leica-TCS-SP8 MP multiphoton excited fluorescence microscope (Leica Microsystem, Milton Keynes, UK). Images were captured using internal spectral hybrid detectors with a gain of around 100. The emission was detected around 472 nm. The images were analysed using Leica LAS-X life science software. 3D images were reconstructed using the same software. 

##### In vitro antifungal activity of AMP-B encapsulated within dissolving MNs

The antifungal activity of AMP-B encapsulated dissolving MN arrays was tested against *Candida albicans* (NCTC 3179) and *Aspergillus niger* (CABI 017,454) by using the agar diffusion method since these genera are common causative agents of fungal infection [39]. In brief, 1 mL of activated fungal growth, either *Candida albicans* (1.5–5 × 10^6^ CFU/mL) or *Aspergillus niger* (2–3 × 10^4^ CFU/mL), was pipetted into the sterile plastic plate, and 15 mL, of sterile molting SDA, 1% agar was poured into the same plate and moved gently for good mixing and dispersing of fungal suspension with medium, and the plate was left to solidify. The MN arrays were firstly separated from the baseplate by a sheet of aluminium foil to avoid passing any extra amount of drug from baseplate to culture medium and keep the source of the drug from dissolving MN arrays only. The arrays of MN were then applied on the surface of inoculated plates for 1–2 min and removed after they were dissolved inside the inoculated medium; the treated plates were incubated at 37 °C for 24 h. The AMP-B suspension was prepared as the same AMP-B concentration that loaded MNs as a positive control. Then, a well was made in the middle of the seeded plate by using a sterile cork borer (6 mm) to pipet a drug suspension. The untreated plate was represented as a negative control. The diameter of the inhibition zone was measured in mm, and triplicates were conducted for each treatment. 

##### Biocompatibility assay

PVP (30%w/w), HA (5%w/w) and a mixture from PVP and HA were dissolved in the medium for cell culture DMEM/F12 supplemented with 10% fetal bovine serum and 1% penicillin/streptomycin. The drug was prepared as a suspension using DMEM/F12. The same concentrations were prepared from drug-free MNs and from AMP-B-loaded MNs by dissolving them in DMEM/F12 medium. All prepared concentrations were then sterilised using ultraviolet (UV) radiation for 15 min. ARPE-19 cells were cultured at a density of 25,000 cells per well into 96-well tissue culture plates (VWR, Leicestershire, England, UK) for 24 h before the assay. The next day, a 200 μL cell culture medium was removed and replaced with the treatments mentioned above and incubated at 37 °C and 5% CO_2_ for a further 24 h. The cell viability was then determined using 10% of the MTS reagent. Following 2 h incubation of the plate at 37 ℃ and 5% CO_2_, absorbance was recorded at 490 nm using a Tecan Sunrise™ plate reader. After subtraction of absorbance values of the blank wells (medium only) from all other absorbance values, percentage cell viability was calculated relative to untreated control wells using the formula given below:

$$\begin{aligned}\mathrm{\% }\;Cell\;& viability\;\\&=\frac{(Abs\;490 \;of\; treated\; cells)-(Abs\;490 \;Blank)}{\left(Abs \;90 \;of \;unteated\; cells\right)-(Abs\;490 \;Blank)}\end{aligned}$$

#### Statistical analysis

The obtained data were analysed statistically using Prism 5.3 (GraphPad, Inc., USA) and Microsoft Excel 11.0 (Microsoft). The mean values of the percentage of cell viability were analysed using an analysis of variance (ANOVA) followed by post hoc Tukey’s multiple comparison tests depending on the number of replicates used, and a *t*-test was used for the antifungal activity. Six replicates were used for treated concentrations and control—a probability of *p* < 0.05 indicated significance.

## Results

### Mechanical and dissolution profiles of MN formulations

**Fig. 1:**
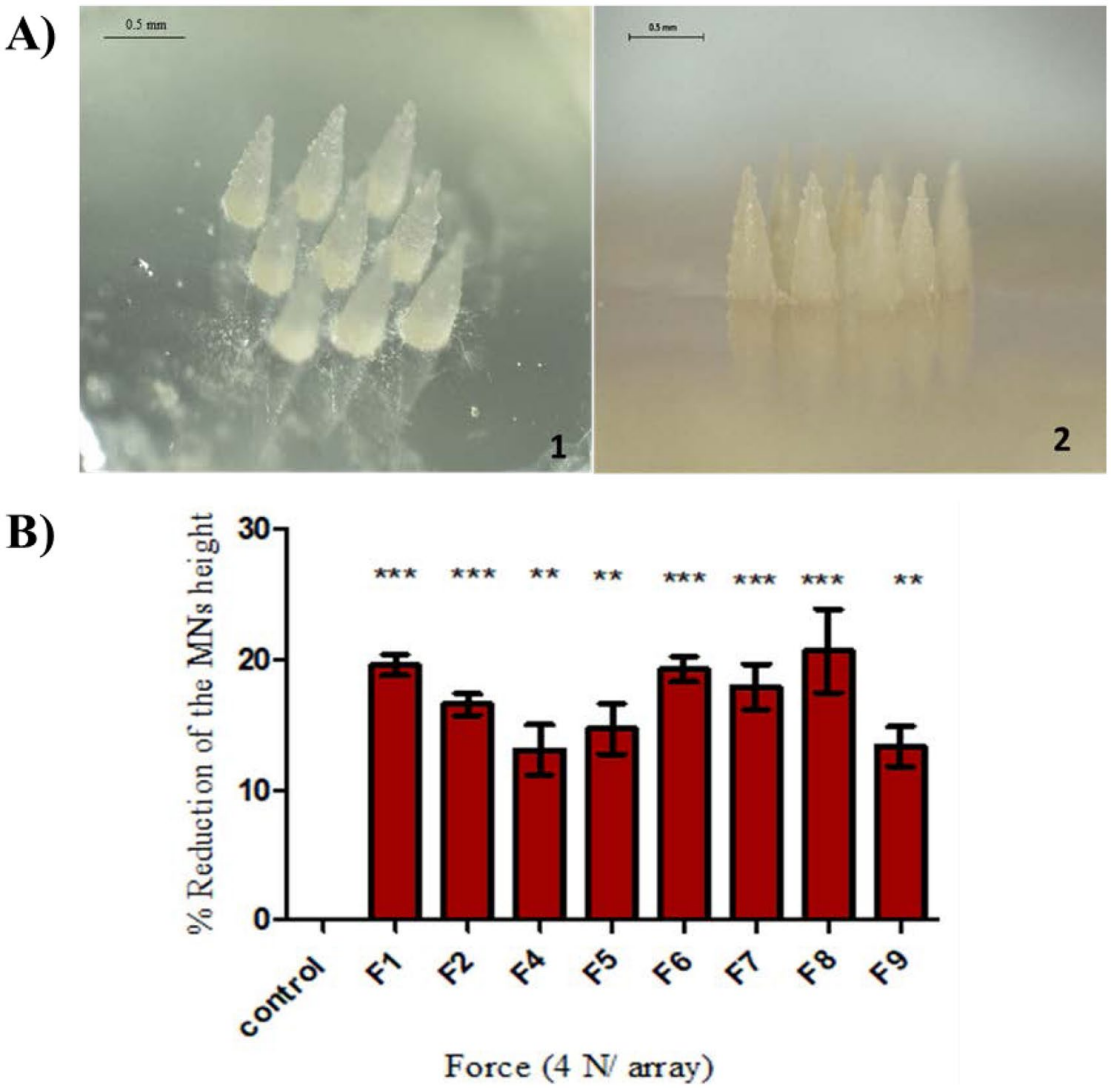
**A** Light microscopic images of polymeric MN arrays produced using silicone mould (1: drug-free MN arrays; 2: AMP-B-loaded MN arrays). **B** Percentage of MN height reduction following 4 N compression force. MN arrays were fabricated from drug-free hydrogels. Data reported as mean ± SD, *n* = 9

MNs were formulated using a mixture of different concentrations of PVP, PVA and HA polymers. Figure 1A shows the images of the formulated MNs following the micromoulding process using a 3 × 3 array silicone mould to produce nine conical MNs (750–800 µm height, 300 µm width, 300 µm interspacing) with sharp tips.

The mechanical strength and dissolution studies were conducted on different formulations by using predefined force (4 N) for compression test and 30 s for dissolution test to examine the durability and solubility of MN arrays’ best formula for further studies. Figure 1B illustrates the effect of the compression test on different formulations. Compression studies revealed that all formulations tolerated the predefined force (4 N) without bending or breaking. F4 formulation was given the best tolerance, with an average height reduction percentage of 13%. F9 formulation had a similar height reduction percentage of 15%, while the F8 formulation had the highest percentage of height reduction (20.6%) compared to other formulations. These results demonstrated that MNs were mechanically strong and were not fractured; instead, they became slightly compressed.

**Fig. 2:**
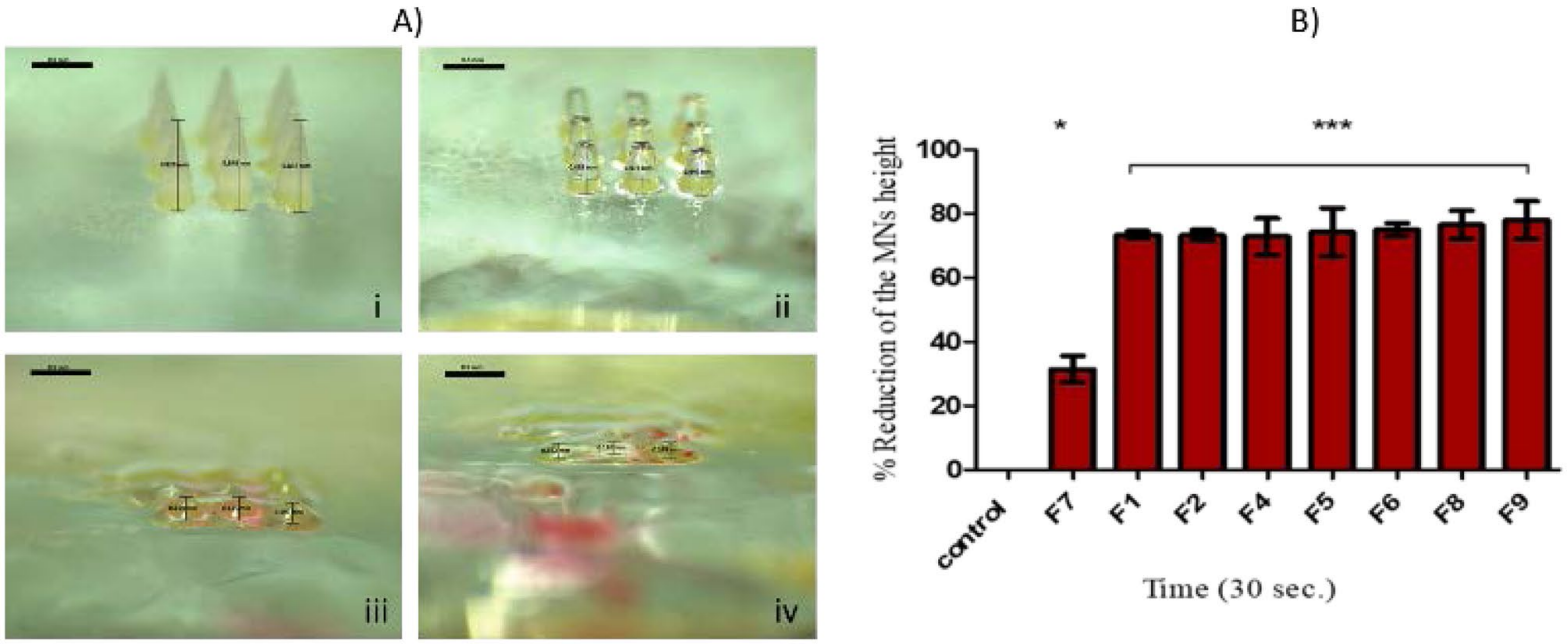
**A** Light microscopy images of polymeric MN arrays before (i) and after 15 s (ii), 20 s (iii) and 30 s (iv) of dissolution. **B** Percentage of the average reduction of MN arrays versus predefined time after insertion into corneal tissue. MN arrays were fabricated from drug-free hydrogels. Data reported as mean ± SD, *n* = 9

A dissolution study was performed to investigate the solubility of the tested formulations following 30 s insertion, the typical time for MN application. As shown in the height reduction of F9 MN tips at 15, 20 and 30 s in Fig. 2A, 30 s was enough to dissolve the MN completely. Results outlined in Fig. 2B that F7 formulation had the lowest solubility, as demonstrated in a lower reduction of the MN height 31.5% compared to F5 (74%), F4 (73%), F8 (76%) and F9 (78%).

Based on both mechanical and dissolutions profiles, F4, F5 and F9 formulations had the best tolerance to compression forces and high dissolution profiles; however, from a physiological point of view, F9 formulation was taken forward for further investigations. Formulations with HA could be more feasible for our ocular delivery studies as HA is a natural polymeric carbohydrate compound, which constitutes part of the ocular tissues has been used in many ophthalmic formulations to provide lubrication and hydration to the eye, to extend drug retention and enhance wound healing [40].

### AMP-B-loaded MN characterisation

AMP-B at concentration 50 mg/10gm was incorporated into the F9 formulation, and the mechanical strength and dissolution profiles were determined. As seen in Table 2, there is no significant difference between the percentage of height reduction for AMP-B-loaded F9 (16.7%) compared to F9 formulation (16%), which confirms that drug loading did not compromise the mechanical strength of the MNs. Similarly, the dissolution profiles for both formulations were greater than 75% following 30 s application on corneal tissue. Evaluation of the drug content of MNs is a critical step to confirm the dosage accuracy and reproducibility. Full drug recovery (100% ± 4.9) from MNs was recorded. The drug content in nine MNs was 6.90 ± 3.51 µg, so each MN had 0.76 µg ± 0.38. Therefore, 30% PVP + 5% HA formulations have an excellent capacity to enclose the drug molecule and re-release it.

**Table 2:** % height reduction of optimised MN formulations with and without AMP-B

Formulation	% reduction of height following compression	% reduction of height following 30 s dissolution
F9	15 ± 5.0	78 ± 10.3
F9 + AMB-B	16.7 ± 6.9	75 ± 5.2

**Fig. 3:**
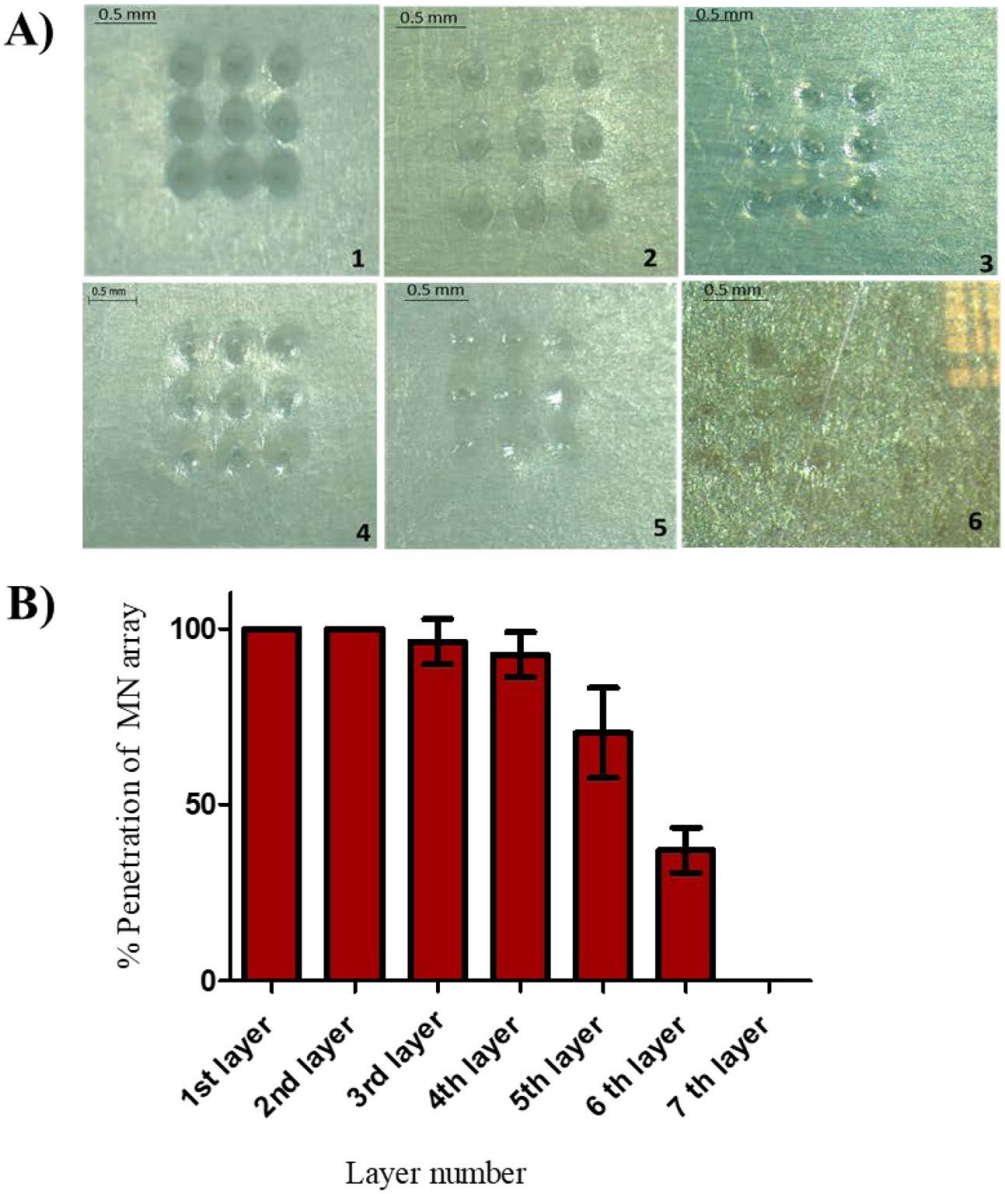
**A** Light microscopic images (1–6 layers) of a top view of the penetrated Parafilm M® layers to visualise the pores due to MN tips penetration in each layer. **B** Percentage of the penetration of MN arrays versus predefined compression force (4 N). MN arrays were fabricated from AMP-B-loaded hydrogels 30% PVP + 5% HA. Data reported as mean ± SD, *n* = 9

AMP-B polymeric MNs were further tested using an artificial folded membrane (Parafilm M®) to mimic the corneal layers. The parafilm sheet’s thickness is 0.13 mm, and corneal tissues are around 0.5–0.8 mm; so, it was folded to get the seven-layer film (0.91 mm) to be adjacent cornea thickness, then the folded film was placed onto poly(ethylene) sheet for support. The insertion test was performed by a texture analyser that was used previously in the compression test at compression mode, and % of MNs insertion into Parafilm layers was determined, as shown in Fig. 3B. Images in Fig. 3A show full penetration in the first and second layers, where the third and fourth layers had excellent penetration, and it was more than 95% for the third layer and 92.59% for the fourth layer. The percentage of penetration decreased to be less than 75% in the fifth layer. In the sixth layer, the percentage was reduced markedly to be 37.03%. However, no penetration was noticed in the seventh layer.

**Fig. 4:**
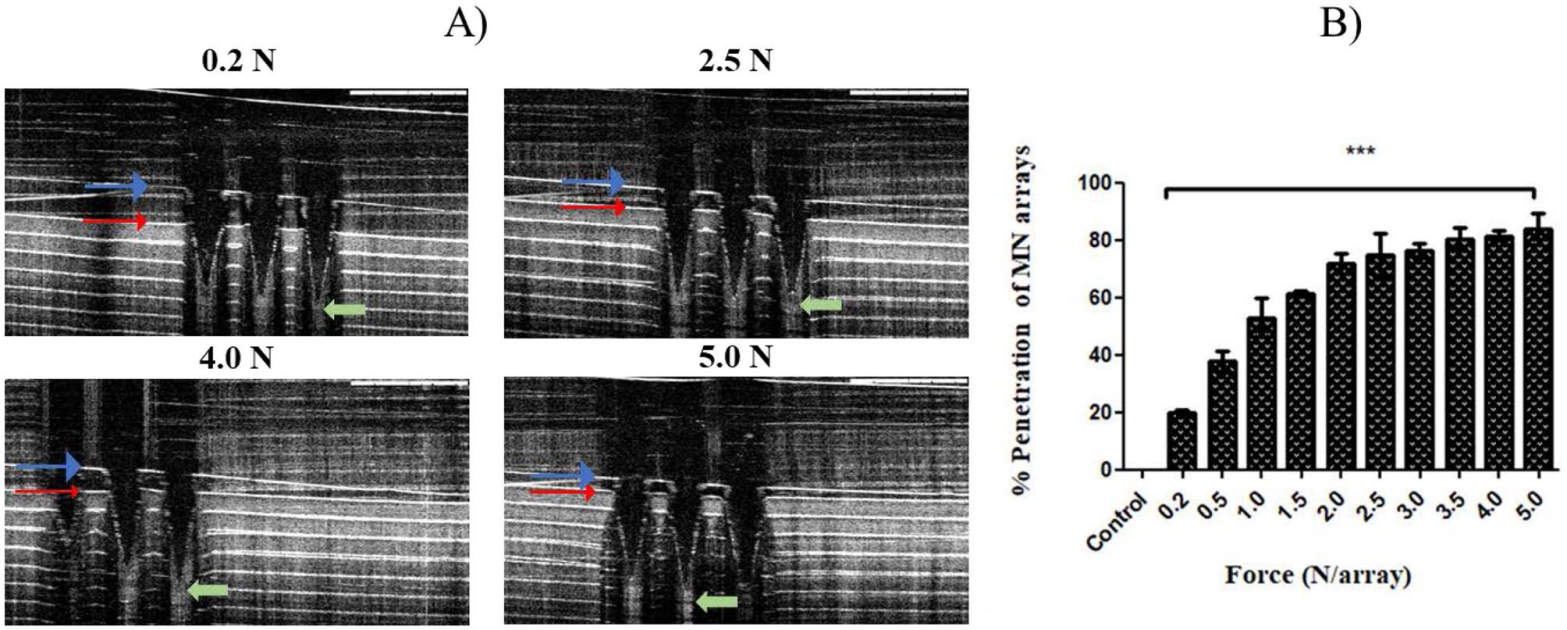
**A** OCT images of loaded AMP-B MN arrays inserted into Parafilm M® layers using several predefined forces (0.2, 2.5, 4.0, 5.0 N). Blue arrow: MN baseplate. Red arrow: First layer of Parafilm M®. The scale bar = 0.5 mm. **B** Percentage of MN arrays penetration versus predefined compression forces. MN arrays were fabricated from AMP-B-loaded hydrogels (30% PVP + 5% HA). Data reported as mean ± SD, *n* = 9, ***p < 0.001 showing a significant difference between compressed MN arrays and control (no MN inserted)

Further testing was conducted using OCT scanning to determine the depth of penetration following the application of a range of predefined forces (0.2, 0.5, 1.0, 1.5, 2.0, 2.5, 3.0, 3.5, 4.0, 5.0 N). As shown in Fig. 4A and B, there is a direct correlation between the depth of inserted MN arrays and applied force.

### In vitro intraocular distribution of AMP-B-loaded MNs

**Fig. 5:**
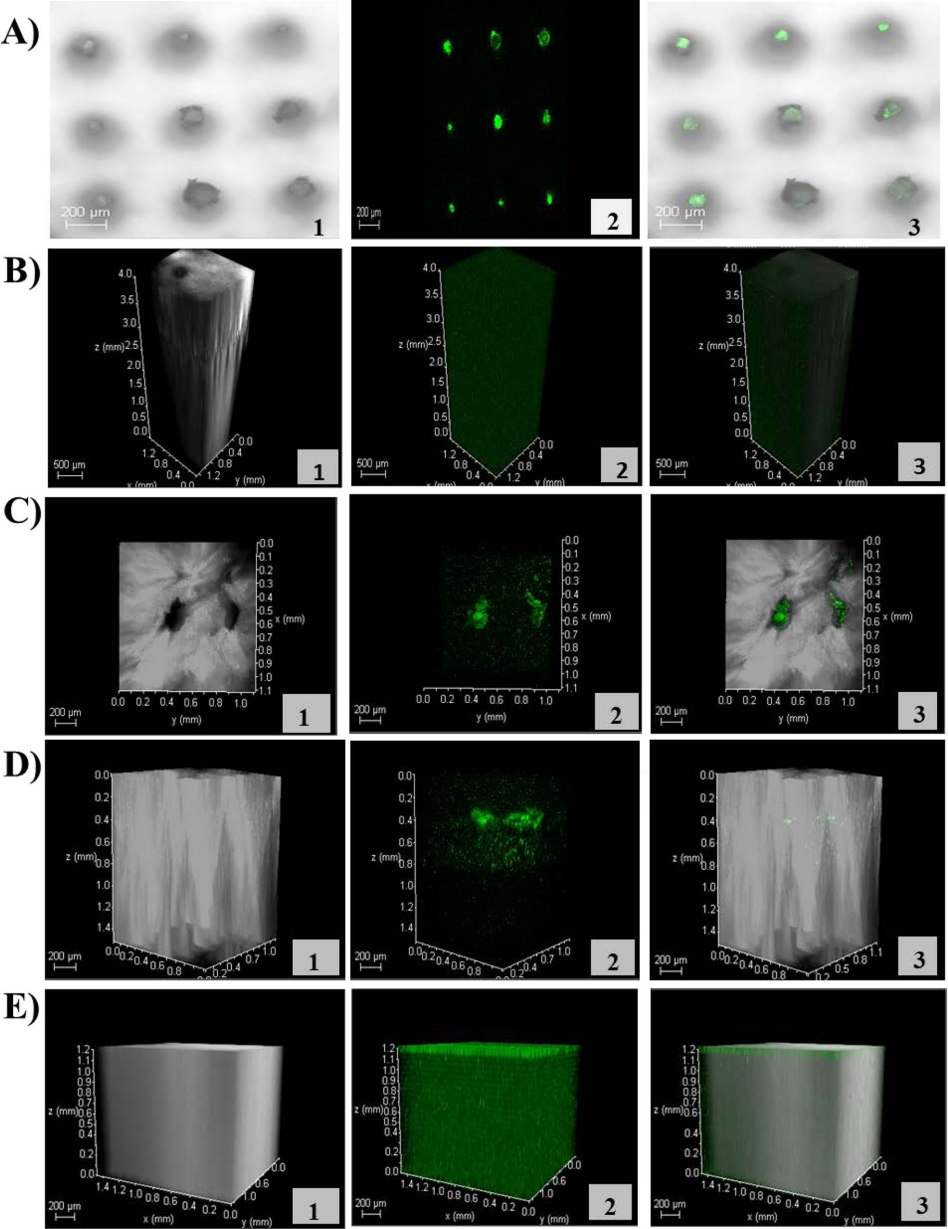
**A** MPM images of polymeric MNs with loaded AMP-B. **B** Three-dimensional MPM images of porcine sclerocorneal tissue. **C** MPM images (top view) of porcine sclerocorneal tissue and AMP-B MN. **D** Three-dimensional MPM images (side view) of porcine sclerocorneal tissue and AMP-B MN. **E** Three-dimensional MPM images of porcine sclerocorneal tissue and AMP-B as a solution. Numbers include (1) the transmitted light channel (grey), (2) the green channel represents auto-fluorescence of AMP-B and (3) merged image of the grey and green images

A multiphoton microscope (MPM) was used to visualise and assess the success of AMP-B delivery within the corneal tissue. Figure 5 includes images of MN arrays using MPM, which are showing the loaded AMP-B MN arrays. In Fig. 5A, it is clear to see the top view of polymeric MN arrays loaded with AMP-B molecules that gives the green auto-fluorescence (Image 2, Fig. 5A). This is confirmed by 3-D imaging of untreated corneal tissue, which is extracted from the porcine eyeball. It is clearly shown that the AMP-B MN array is penetrating the corneal tissue (Fig. 5C).

AMP-B was administrated on the porcine corneal tissue, either loaded in MNs or as a solution. Following 5 min, 3D imaging for AMP-B MN arrays showed traces of the drug on the tissue’s surface and within the corneal tissue, as shown in Fig. 5C. Analysis of 3-D images in Fig. 5D can depict the depth of AMP-B within the corneal layers, usually ranging from 0.4 to 0.5 mm, representing the thickness of the cornea. The green channel image 2 in Fig. 5D reveals AMP-B is delivered at a depth of 0.4 mm, and some of the drug molecules are moved away from the corneal epithelium layer to the bowman’s layer, while no evident penetration was determined when delivered as a solution (Fig. 5E).

### Antifungal activity of AMP-B-loaded MNs

**Fig. 6:**
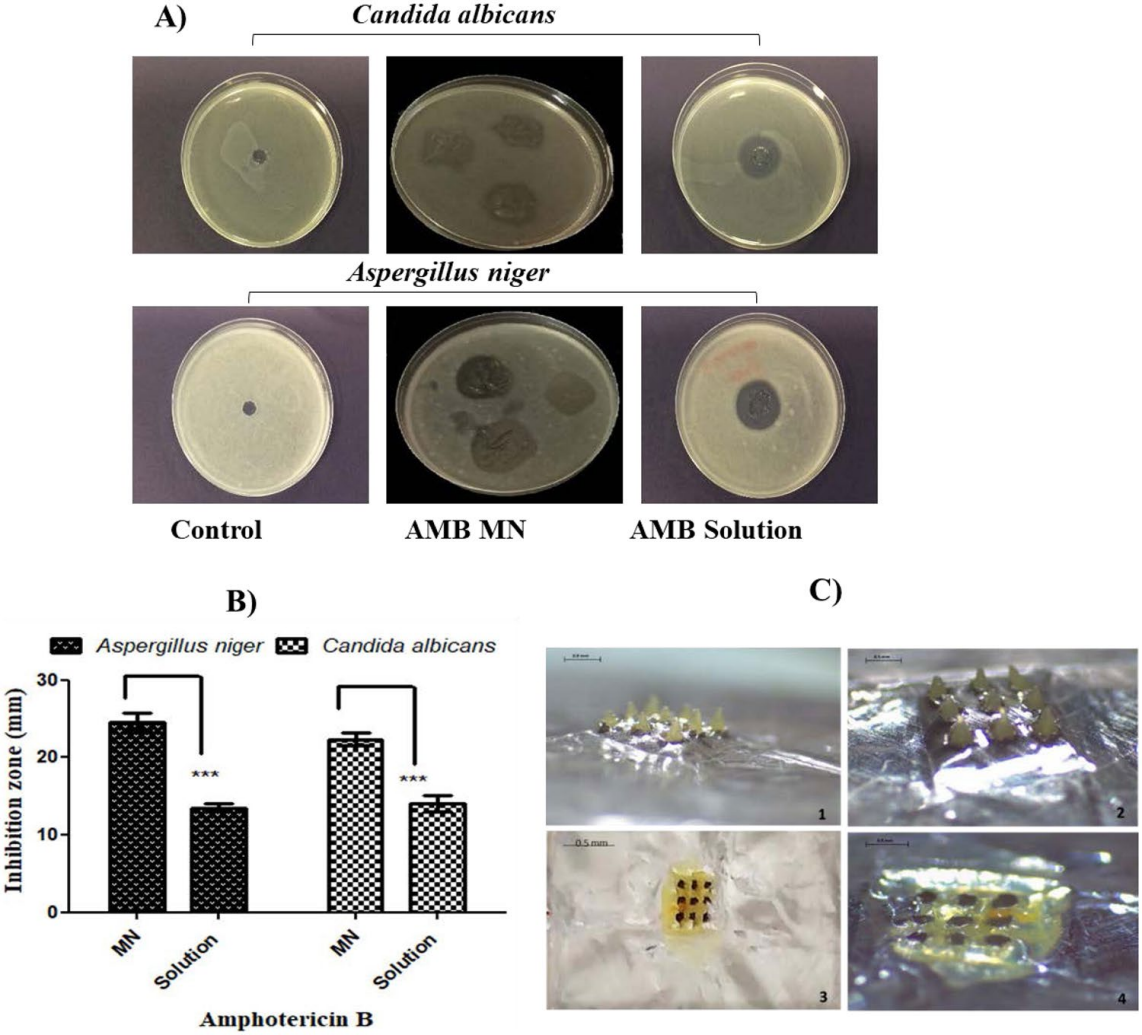
Representative images **A** and graph **B** showing the antifungal activity for AMB MNs performed on the soft SDA against two different types of fungi, *Aspergillus niger* and *Candida albicans*. The results were read after 24 h incubation at 37 °C. Data reported as mean ± SD, *n* = 6, ***p < 0.001. (C) Light microscopic images of MN arrays before application on inoculated medium (1, 2) and after application on inoculated medium (3, 4). The arrays were separated from the baseplate by aluminium foil

Antifungal activity of AMP-B loaded in MNs was studied in 2 common species that cause ocular fungal infection, *Candida albicans* and *Aspergillus niger*. The diameter of the zone of inhibition is shown in Fig. 6. Clear zones of inhibition were obtained. Results revealed a significant antifungal effect of AMP-B encapsulated MNs (*p* < 0.001) compared to its suspension form. These results are indicating the potential antifungal efficacy of prepared AMP-B MNs. AMP-B encapsulated MNs were showing a rapid dissolution (2 min) following application on the inoculated medium and were all dissolved completely inside the SDA medium (Fig. 6C).

### In vitrobiocompatibility study

**Fig. 7:**
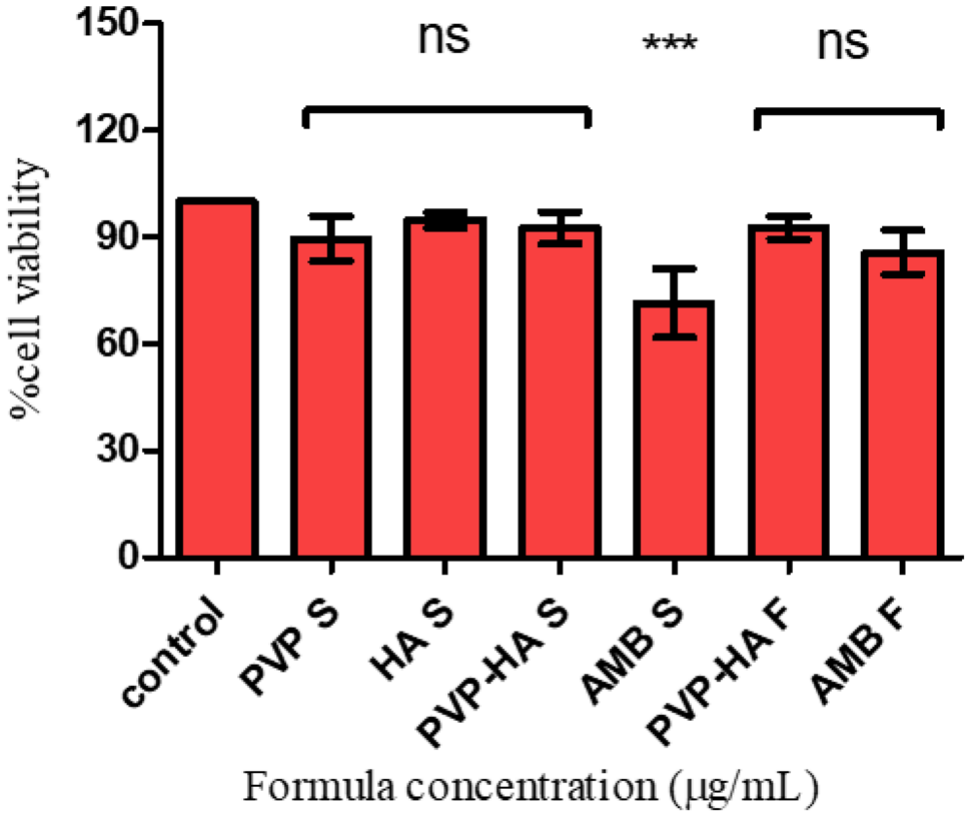
Percentage viability of human retinal pigment epithelium cells (ARPE-19) following 24 h exposure to different polymers and AMP-B (AMB) as solutions and after formulation. Results are reported an average of 6 replicates. ns = no significant effect (*p* > 0.05) between formula and control (non-treated), *** *p* < 0.005

AMP-B formulation’s cytotoxicity effect was investigated using ocular cell lines, adult retinal pigment epithelial 19 (ARPE-19). It can be seen from Fig. 7 that the selected polymers (PVP and HA) had high biocompatibility, as demonstrated by high cell viability. The average cell viability for these polymers was more than 90%. A significant reduction (*p* < 0.001) in cell viability was seen following cell treatment with a free drug (71.45 ± 9.69%), while the formulated AMP-B had no significant decrease in cell viability compared to the with untreated cells (85.74 ± 6.23%).

## Discussion

Safe and effective drugs become an essential need for increasing the rate of morbidity and mortality due to microbial infections. It was estimated that mortality rates from fungal infections alone are over 1.5 M annually [3]. Fungal infection is a devastating microbial cornea infection that could lead to blindness if it is not diagnosed and treated correctly. Topical AMP-B is considered the treatment of choice for fungal infection. However, it has serious side effects, particularly dose-limiting nephrotoxicity [41]. The current topical formulations of AMP-B contain deoxycholate, necessary to solubilise the highly hydrophobic AMP-B, render their instillation painful and lead to poor compliance [42]. Although less toxic lipid formulations of AMP-B can be used in patients unresponsive to conventional AMP-B, the ocular bioavailability of some liposomal drugs has been proven to be equal to that of non-liposomal ones [43]. Therefore, there is an urgent need to develop a formulation that would be better tolerated in the eye, without using an irritating surfactant, and more importantly, improve penetration in the ocular layers and reach its site of action and release the AMP-B. MNs are emerging as a minimally invasive method to enhance ocular drug delivery with no irritation [29]. However, most studies had either used model drugs [44, 45] or drug-like pilocarpine [46]. Only one study was published recently, which used liposomal AMP-B, and pure AMP-B encapsulated PVP/PVA MNs to treat ocular fungal infection [35]. However, liposomal formulation’s lipidic nature could affect mechanical strength, particularly with high drug loading. Apart from this, the liposomal formulation could also increase the cost of the overall final MN patch. Thus, direct loading of the pure drug within the dissolving MN arrays offers an economical and scalable opportunity.

In this study, we developed a biodegradable MN system for ocular delivery of AMP-B. Results from the study clearly showed that MNs made from PVP/HA (30%:5%w/w) were able to encapsulate the drug and release it upon contact with ocular tissue. Both PVP and HA were shown previously in the literature to be biocompatible. HA is a natural material that is rich in the anterior part of the eye [47]. Within the ocular drug delivery, both PVP and HA have been used previously as ophthalmic inserts [48], in situ gels [49], and recently using MNs [26, 29]. In the present study, PV HA formulation was incorporated with AMP-B to formulate MNs. The data obtained from the present study clearly illustrates that the MN arrays can be produced with excellent reproducibility, strength and faster dissolution rate with 100% drug recovery. Chosen formulation of PVP/HA for MN fabrication was proven to fully penetrate through multiple layers of Parafilm M® without breaking or bending. This was attributed to the PVP polymer, which harnessed to strengthen the MNs due to its unique chemical structure (ring and hydrogen bonds) [50]. Furthermore, these MNs showed high solubility, which is an advantage when MNs were inserted. For the first time, we have demonstrated the use of a multiphoton microscope to visualize the AMP-B-loaded MN insertion in the corneal tissue and successfully deposited the AMP-B within the corneal tissue. Charvalos et al. [17] have demonstrated that using PVP complexes for AMP-B formulation significantly reduced its cytotoxicity, which was parallel with this study. Both PVP and HA successfully decreased AMP-B cytotoxicity, comparing with free drug. More interestingly, the biocompatible formulations preserved the antifungal activity of AMP-B as demonstrated by significant inhibition of fungal growth after 24 h of incubation.

The promising results of this study have proven the study’s concept that pure AMP-B can be effectively delivered into the corneal tissue using fast-dissolving/biocompatible MN arrays composition and AMP-B can exhibit its antifungal activity at the site of application. The overriding advantages of the site-specific minimally invasive delivery system we have developed here have potential for localized and long-term treatment of fungal infections in the eye, which could potentially enhance the efficacy of marketed antifungal ocular therapy. Even though we have seen encouraging in vitro results from the AMP-B/MNs, the in vivo ocular pharmacokinetics studies must be investigated to ensure clinically relevant drug levels are achieved. Additionally, in vivo efficacy studies must also be performed to evaluate this approach’s antifungal efficacy in animal models.

## Conclusions

This study shows the feasibility of ocular delivery of the poorly soluble AMP-B. We successfully demonstrated the potential use of simple dissolvable PVP/HA MNs for intracorneal AMP-B delivery. The combination of low toxicity, faster dissolution, full penetration through corneal tissues with 100% drug recovery and, more importantly, retained antifungal activity would considerably increase the therapeutic index for the antifungal drug in treating ocular fungal infection. Further studies are to be undertaken to optimise the MN system for pre-clinical testing.
